# Inhibition of Nitric Oxide Synthesis Prevents the Effects of Intermittent Social Defeat on Cocaine-Induced Conditioned Place Preference in Male Mice

**DOI:** 10.3390/ph17091203

**Published:** 2024-09-12

**Authors:** María Ángeles Martínez-Caballero, María Pilar García-Pardo, Claudia Calpe-López, María Carmen Arenas, Carmen Manzanedo, María Asuncion Aguilar

**Affiliations:** 1 Neurobehavioural Mechanisms and Endophenotypes of Addictive Behavior Research Unit, Department of Psychobiology, University of Valencia, 46010 Valencia, Spain; maria.a.martinez-caballero@uv.es; 2 Department of Psychology and Sociology, Faculty of Social Sciences, University of Zaragoza, 50009 Teruel, Spain; magarpar@unizar.es; 3 Institute of Psychopharmacology, Central Institute of Mental Health, Medical Faculty Mannheim, University of Heidelberg, 68167 Mannheim, Germany; claudia.calpelopez@zi-mannheim.de; 4 Department of Psychobiology, University of Valencia, 46010 Valencia, Spain; carmen.arenas@uv.es (M.C.A.); carmen.manzanedo@uv.es (C.M.)

**Keywords:** cocaine, conditioned place preference, *mice*, nitric oxide synthase, social defeat stress, anxiety-like effects, depression-like effects

## Abstract

We have previously observed that *mice* exposed to social defeat stress are more sensitive to cocaine in the conditioned place preference (CPP) paradigm. In this context, it has been suggested that the nitric oxide (NO) pathway plays a role in the effects of stress. The present study evaluates the role of a neuronal NO synthase (nNOS) inhibitor (7-nitroindazole, 7-NI) in the short- and long-term behavioural effects of intermittent social defeat (ISD). Four groups of *mice* were employed for the study: a control group and three stressed groups, one treated with vehicle and two treated with 7-NI (7.25 or 12.5 mg/kg). After the last episode of defeat, *mice* were tested in the elevated plus maze (EPM), social interaction, object recognition and tail suspension tests. Three weeks later, *mice* were conditioned with cocaine (1 mg/kg). Stressed *mice*, irrespective of the treatment received, showed anxiety in the EPM, presented a deficit of social interaction and spent less time immobile in the tail suspension test. However, only stressed *mice* treated with vehicle developed CPP. Thus, although 7-NI did not modify the short-term behavioural effects of ISD, it prevented ISD-induced potentiation of the rewarding properties of cocaine in adulthood. These results support a specific role of nNOS in the effects of social stress on drug reward.

## 1. Introduction

Environmental factors, such as stress exposure, can make individuals more vulnerable to developing drug abuse [1,2,3]. In previous studies, we have observed that adult male *mice* exposed to intermittent social defeat (ISD) during late adolescence (post-natal day (PND) 47, 50, 53 and 56) are more sensitive to the rewarding effects of cocaine on the conditioned place preference (CPP) paradigm [4,5,6,7]. In addition, we have seen that ISD induces several short-term behavioural alterations that could be related with the subsequent resilience or vulnerability of *mice* to the development of cocaine CPP, such as a decrease in the number of entries and time spent in the open arms of the elevated plus maze (EPM), social avoidance in the social interaction test, greater immobility in the tail suspension test, and a reduction of grooming in the splash test [5].

Several peripheral and central stress responses, including autonomic or hypothalamic–pituitary–adrenal (HPA) responses and structural and functional brain alterations (decreased prefrontal functioning and increased limbic–striatal activation), have been associated with the pathophysiology of substance use disorders [8]. Regarding the neurobiological substrates of the effects of ISD on cocaine reward, we have observed that adult *mice* exposed to ISD during late adolescence show decreased expression of brain-derived neurotrophic factor (proBDNF) in the ventral tegmental area (VTA) and its receptor tropomyosin receptor kinase B (TrkB) in the nucleus accumbens (NAcc) [9]. In addition, we have seen how the enhanced response to cocaine after social stress is reversed by antagonism of the corticotropin-releasing factor type 1 (CRF1) [10] and N-methyl-D-aspartate (NMDA) glutamate receptors [4].

Using a different protocol of SD stress (four episodes of SD on alternate days), we have previously observed that NMDA and AMPA (α-amino-3-hydroxy-5-methyl-4-isoxazolepropionic acid) glutamate receptors and neuronal nitric oxide synthase (nNOS), a downstream signal molecule of NMDA receptor activation, are implicated in the effects of SD on the rewarding properties of the psychostimulant drug MDMA (3,4 methylenedioxymethamphetamine) in *mice* [11,12]. In particular, exposure to an episode of defeat immediately before each conditioning session with MDMA (PND 54, 56, 58 and 60) blunted the rewarding effects of this drug in the CPP paradigm, and this effect was prevented by administration of the NMDA antagonist memantine [11] and the nNOS inhibitor 7-nitroindazole (7-NI) [12]. Moreover, exposure to this protocol of SD decreased the expression of several subunits of NMDA and AMPA receptors—mainly GluN1 and GluA1—in the striatum and hippocampus [11] and increased nitrite levels in these structures [12] 48 h after the last episode of defeat. Considered as a whole, these results demonstrate that glutamatergic–nitric oxide plasticity is involved in the effects of SD stress on MDMA reward.

The role of NO in other behavioural consequences of social stress, such as depression- and anxiety-like symptomatology, has only been studied using the protocol of chronic SD stress (CSDS), in which experimental animals are exposed to daily episodes of defeat on 10 consecutive days and are in sensory contact with opponents for a 24 h interval between each episode. Social avoidance and depression-like behaviours observed in *mice* exposed to CSDS are known to be mediated by nNOS enzyme activity and NO production, as CSDS increases the expression of nNOS in the shell of the NAcc [13]. Similarly, increased anxiety-like behaviour induced by CSDS is accompanied by the activation of nNOS-containing neurons in the right (but not left) medial prefrontal cortex [14].

In light of the above evidence, the aim of the present study was to evaluate the role of the NO pathway in the behavioural effects of ISD. With this purpose in mind, we tested whether the nNOS enzyme inhibitor 7-NI can prevent the short-term effects of ISD exposure in young *mice* undergoing the EPM, social interaction, object recognition and tail suspension tests 24/48 h after the last defeat episode (anxiety- and depression-like symptoms, cognitive deficits and stress hyperreactivity). In addition, we explored whether 7-NI can block the long-term effects of ISD on cocaine reward, i.e., the potentiation of the rewarding effects of cocaine observed in adult *mice* exposed to ISD during late adolescence.

## 2. Results

### 2.1. Behavioural Analysis during Episodes of Defeat

The data of the time spent in the different behavioural categories and latencies to show these behaviours are shown in Table 1. ANOVA of the threat data showed that the variables labelled as days [F(1,31) = 4.696; *p* < 0.05] and treatment [F(2,31) = 4.694; *p* < 0.05] were significant, but not their interaction. *Mice* spent more time under threat from the opponent in the first than in the last episode of defeat (*p* < 0.05), and the 7-NI12 + SD group spent less time under threat from the opponent than the vehicle + SD group (*p* < 0.05). In addition, ANOVA of the latency of avoidance/flee behaviour revealed that the variable labelled treatment was significant [F(2,31) = 4.349; *p* < 0.05] and that *mice* in the 7-NI12 + SD group showed lower latency to avoid or flee the opponent than those in the vehicle + SD group (*p* < 0.05). As data for latency of avoidance/flee in the first episode of defeat and of latency of defence/submission in the fourth episode have unequal variances we performed ANOVA–Welch, the results of which are not significant.

### 2.2. Role of 7-NI in the Short-Term Effects of ISD

**Figure 1:**
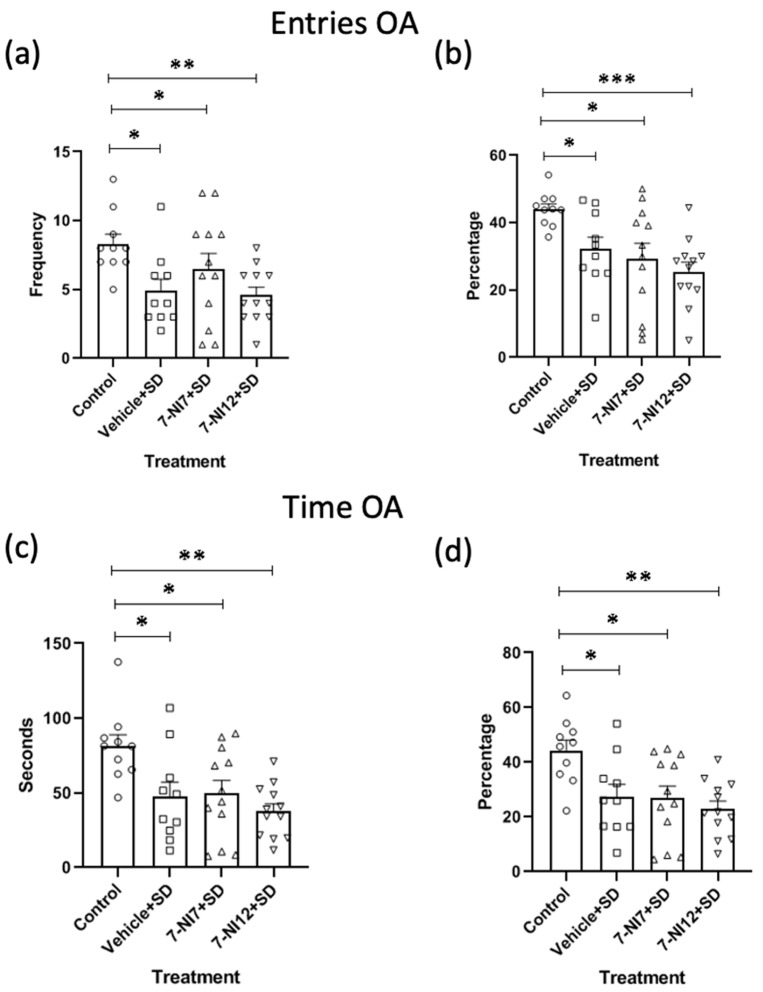
Role of 7-nitroindazole in the effects of intermittent social defeat (SD) on the behaviour of *mice* in the open arms (OA) of the elevated plus maze (EPM). Four groups of *mice* were used according to the treatment received: Control (vehicle + no stress), vehicle + SD (vehicle 90 min before exposure to each episode of social defeat), 7-NI7 + SD (7.25 mg/kg of 7-nitroindazole 90 min before exposure to each episode of social defeat) and 7-NI12 + SD (12.5 mg/kg of 7-nitroindazole 90 min before exposure to each episode of social defeat). The animals’ behaviours in the EPM were evaluated on PND 57 (24 h after the last episode of social defeat). Bars represent the mean (±SD) number of entries into the OA (**a**), percentage of entries into the OA (**b**), time spent in the OA (**c**) and percentage of time spent in the OA (**d**) for each group. * *p* < 0.05, ** *p* < 0.01, *** *p* < 0.001, significant difference with respect to the control group.

Regarding the elevated plus maze (EPM), the ANOVAs revealed that the values for entries in open arms [EOA F(3,40) = 3.801; *p* < 0.05], percentage of entries in open arms [%EOA F(3,40) = 5.526; *p* < 0.01], time in open arms [TOA F(3,40) = 5.521; *p* < 0.01] and percentage of time in open arms [%TOA F(3,40) = 5.452; *p* < 0.01] were significant. Post-hoc comparison of the treatment variable showed a reduction in these measurements in all stressed *mice* in comparison with the control group, irrespective of whether they received treatment with vehicle or 7-NI. In particular, *mice* treated with vehicle + SD and 7-NI7 + SD differed from controls (ps < 0.05) in EOA, %EOA, TOA and %TOA (Figure 1a–d, respectively), and the differences between *mice* treated with 7-NI12 + SD and controls were more significant (*p* < 0.01 for EOA, TOA and %TOA and *p* < 0.001 for %EOA). ANOVAs for latency to enter into the open arms (LOA) and number of total entries into the arms (TotalE) did not reveal significance.

**Figure 2:**
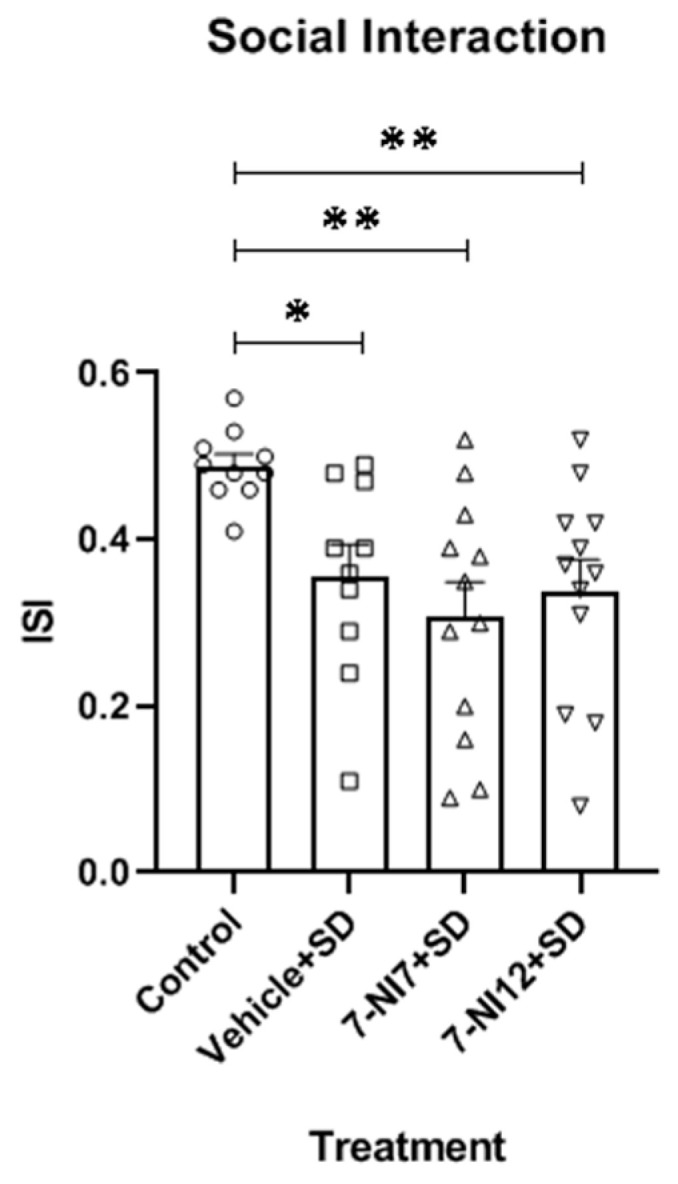
Role of 7-nitroindazole in the effects of intermittent social defeat (SD) on the behaviour of *mice* in the social interaction test. Four groups of *mice* were used according to the treatment received: Control (vehicle + no stress), vehicle + SD (vehicle 90 min before exposure to each episode of social defeat), 7-NI7 + SD (7.25 mg/kg of 7-nitroindazole 90 min before exposure to each episode of social defeat) and 7-NI12 + SD (12.5 mg/kg of 7-nitroindazole 90 min before exposure to each episode of social defeat). The animals’ behaviours were evaluated on PND 57 (24 h after the last episode of social defeat). Bars represent the mean (±SD) index of social interaction (ISI) in each group. * *p* < 0.05, ** *p* < 0.01, significant difference with respect to the Control group.

ANOVA of the social interaction data (Figure 2) also confirmed significance [F(3,40) = 4.802; *p* < 0.01]. Post-hoc comparison of the treatment variable revealed a reduction of the index of social interaction (ISI) in comparison with controls among *mice* in the vehicle + SD, 7NI7 + SD and 7-NI12 + SD groups (*p* < 0.05, *p* < 0.01 and *p* < 0.01, respectively).

**Figure 3:**
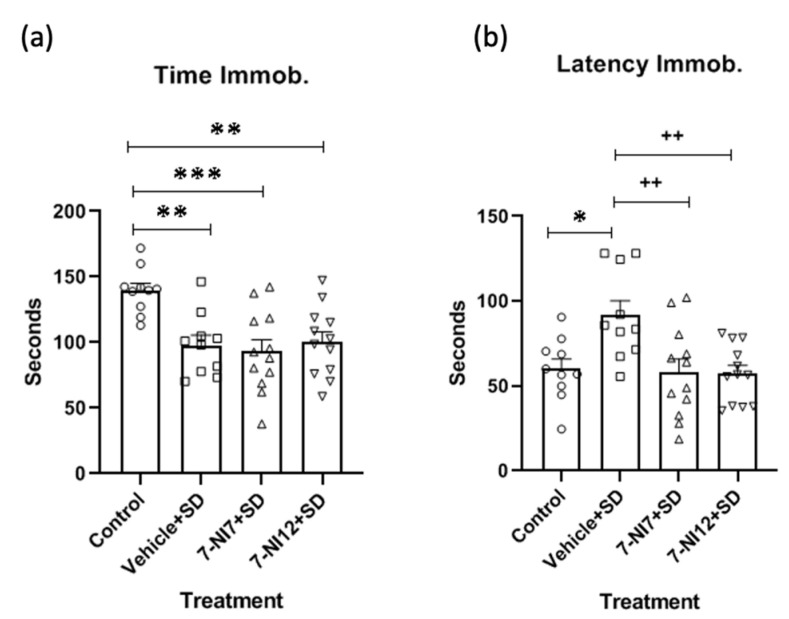
Role of 7-nitroindazole in the effects of intermittent social defeat (SD) on the behaviour of *mice* in the tail suspension test. Four groups of *mice* were used according to the treatment received: Control (vehicle + no stress), vehicle + SD (vehicle 90 min before exposure to each episode of social defeat), 7-NI7 + SD (7.25 mg/kg of 7-nitroindazole 90 min before exposure to each episode of social defeat) and 7-NI12 + SD (12.5 mg/kg of 7-nitroindazole 90 min before exposure to each episode of social defeat). The animals’ behaviours were evaluated on PND 58 (48 h after the last episode of social defeat). Bars represent the mean (±SD) time (in seconds) spent immobile (Time Immob.) (**a**) and the latency (in seconds) to show immobility (Latency Immob.) (**b**) in each group. * *p* < 0.05, ** *p* < 0.01, *** *p* < 0.001, significant difference with respect to the control group. ++ *p* < 0.01, significant difference with respect to the vehicle + SD group.

In relation to the tail suspension test (TST), ANOVA of the time spent immobile (Figure 3a) showed that values were significant [F(3,40) = 7.261; *p* < 0.001]. Post-hoc comparison of the treatment variable revealed that *mice* in the vehicle + SD, 7NI7 + SD and 7-NI12 + SD groups spent less time immobile than controls (*p* < 0.01, *p* < 0.001 and *p* < 0.01, respectively). ANOVA of the latency to become immobile data (Figure 3b) also confirmed significance [F(3,40) = 5.714; *p* < 0.002]; however, in this case, post-hoc comparison revealed that all the groups differed from the vehicle + SD group (*p* < 0.05 vs. control; ps < 0.01 vs. 7-NI7 + SD and 7-NI12 + SD groups).

ANOVA of the object recognition data did not show values to be significant (Supplementary Figure S1).

### 2.3. Role of 7-NI in the Long-Term Effects of ISD

**Figure 4:**
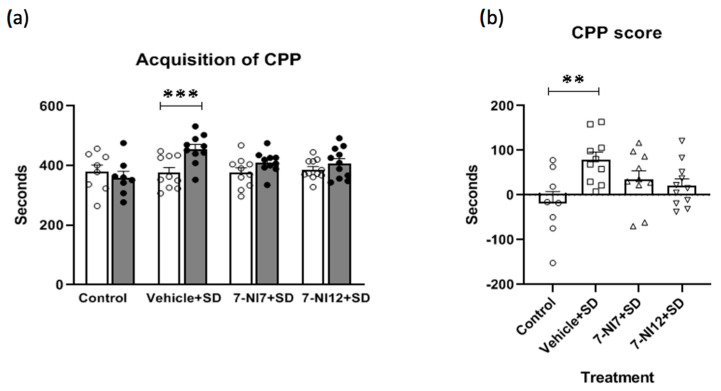
Role of 7-nitroindazole in the effects of intermittent social defeat (SD) on the behaviour of *mice* in the conditioned place preference (CPP) paradigm. Four groups of *mice* were used according to the treatment received: Control (vehicle + no stress), vehicle + SD (vehicle 90 min before exposure to each episode of social defeat), 7-NI7 + SD (7.25 mg/kg of 7-nitroindazole 90 min before exposure to each episode of social defeat) and 7-NI12 + SD (12.5 mg/kg of 7-nitroindazole 90 min before exposure to each episode of social defeat). The CPP procedure was initiated on PND 77 (3 weeks after the last episode of social defeat). (**a**) Acquisition of CPP. Bars represent the mean (±SD) time (in seconds) spent in drug-paired compartment before conditioning (pre-C, white bars) and after conditioning (post-C, black bars) with 1 mg/kg of cocaine. *** *p* < 0.001, significant difference in the time spent in pre-C vs. post-C. (**b**) CPP score. Bars represent the mean (±SD) CPP score (time spent in post-C minus time spent in pre-C in the drug-paired compartment). ** *p* < 0.01, significant difference with respect to the control group.

ANOVA of the CPP score (Figure 4a) confirmed significance [F(3,35) = 4.157, *p* < 0.05]. Post-hoc comparison of the treatment variable revealed that only the vehicle + SD group obtained a higher CPP score than the control group (*p* < 0.01). In addition, ANOVA of the time spent in the drug-paired compartment (Figure 4b) revealed that the days variable [F(1,35) = 9.259, *p* < 0.01] and the interaction days X group variable [F(3,35) = 4.157, *p* < 0.05] were significant, while the treatment variable was not significant. Post-hoc comparison of the interaction variable showed that only stressed *mice* treated with vehicle (and not those treated with 7-NI) developed CPP (*p* < 0.001; significant difference in the time spent in the drug-paired compartment in pre-C vs. post-C in the vehicle + SD group).

### 2.4. Correlations between Behavioural Measurements

Significant Pearson correlations are shown in Table 2 and Table 3. Among the results, it is notable that preference for the open arms of the EPM correlated with several behaviours in the fourth episode of defeat; in particular, TOA and %TOA correlated positively with attack and avoidance/flee, but negatively with defence/submission (ps < 0.05). ISI negatively correlated with threat in the first episode of defeat and attack in the fourth episode of defeat (ps < 0.05). In addition, immobility in the TST and CPP score correlated positively with defence/submission in the fourth episode of defeat (ps < 0.05), while positive correlations were observed between immobility in the TST and ISI (*p* < 0.01), and between CPP score and LOA (*p* < 0.01).

## 3. Discussion

The results of the present study indicate that administration of the selective nNOS inhibitor 7-NI does not modify the short-term behavioural effects of ISD, such as the presence of anxiety-like symptoms in the EPM, the deficit of social interaction and hyperreactivity in a stressful situation (TST). In contrast, 7-NI is able to prevent the increase in latency of immobility in the TST that we observed in defeated *mice*. More importantly, administration of 7-NI before each experience of defeat during late-adolescence prevents ISD-induced potentiation of the rewarding properties of cocaine in adulthood.

Administration of 7-NI did not induce effects on the behaviour of *mice* during social defeat episodes, with the exception of a decrease in the latency when avoiding or fleeing the opponent (which consequently showed lower levels of threat) observed in *mice* treated with the dose of 12.5 mg/kg. Although this behavioural trait of *mice* treated with the high dose of 7-NI could be interpreted as a more active coping strategy (which could reduce the short-term negative consequences of social defeat, such as anxiety- or depression-like symptoms), this idea is not supported by the results observed. There are no differences between groups of defeated *mice* regarding other behaviours frequently associated with active coping, such as higher time spent in avoidance/fleeing or lower time spent in defence/submission. In addition, *mice* of all groups exposed to ISD (irrespective of the treatment with vehicle or any dose of 7-NI) showed a similar behavioural response in every test performed shortly after the last episode of defeat (EPM, social interaction, TST and object recognition).

Exposure to ISD during late adolescence reduced the number of entries and time spent in the open arms of the EPM, as well as the percentages of these measures, thus indicating the presence of anxiety-like symptoms in these defeated *mice*, in line with previous studies carried out in our laboratory [5,6]. We expected that inhibition of nNOS with 7-NI would prevent the anxiety-like effects of ISD, as this compound has anxiolytic properties in animal models of anxiety [15], and we have previously observed an increase in nitrite levels in the striatum and hippocampus after SD [12]. In addition, other protocols of repeated stress have been shown to activate the NO pathway in *mice*; for example, chronic unpredictable stress increased NOS activity in the prefrontal cortex and hippocampus [16], while CSDS activated nNOS-containing neurons in the right medial prefrontal cortex and reduced open-arm exploration [14]. However, in the present study, 7-NI failed to produce anxiolysis in defeated *mice*, in line with studies in which similar results were obtained with acute (6 h) immobilization-induced restraint stress, a protocol that also increases nitrite levels [17,18]. 7-NI was not found to induce an anxiolytic effect in the EPM in restrained *mice* [17,18,19,20] and failed to block the hippocampal NOS activation induced by exposure to a protocol of stress–restress [21]. Authors of these papers proposed that overproduction of NO following stress is likely to involve iNOS and not nNOS, as they observed that the iNOS inhibitor aminoguanidine was effective in reducing anxiety in stressed animals [17,18,19,20,21]. In addition, it has been suggested that the behavioural effects of NOS inhibitors can vary in stressed animals. For example, 7-NI produces an anxiolytic effect in unstressed *mice*, but not in stressed *mice* [15,17,18,19,20], which can present increased anxiety after 7-NI administration [22].

As we have previously observed, ISD led to avoidance of social exploration in our *mice* [5,7]. We expected 7-nitroindazole to reverse this effect of ISD, as nNOS has been implicated in the pathophysiology of depressive disorders [13,23,24] and overexpression of nNOS in NAcc-induced social avoidance [13]. Moreover, administration of the nNOS-specific inhibitor L-VNIO in the NAcc prevented the deficit of social interaction induced by CSDS [13]. Although the effects of 7-NI on social avoidance induced by ISD have not been studied previously, this compound has been shown to reverse the reduction in social interaction observed in *Shank3* mutant *mice*, a *mouse* model of autism, when administered at 80 mg/kg 7 days before SIT [25,26]. Conversely to our hypothesis, we have observed in the present study that administration of 7-NI before each episode of defeat does not prevent the social avoidance induced by ISD. Differences in the protocol used to induce stress (ISD vs. CSDS) and in the nNOS inhibitor employed (7-NI vs. L-VNIO) could explain the divergence between previous results [13] and the present data.

ISD reduced the time of immobility in the TST, in accordance with previous studies performed in our laboratory [5,6], and 7-NI did not reverse this effect. In line with the conventional interpretation of immobility in the TST as behavioural despair, several studies have indicated a role of nNOS in depression-like behaviours. nNOS overexpression in the NAcc of *mice* is enough to increase immobility in the TST and reduce sucrose preference [13]. In addition, the increase in immobility induced by chronic mild stress (CMS) in the TST and forced swim test (FST) was shown to be reversed by the systemic administration of 7-NI [23], and the same effects were observed after intra-NAcc administration of L-VNIO to *mice* exposed to CSDS [13]. Conversely, 7-NI (25 mg/kg, 30 min before TST) did not reverse the increase in the time spent immobile in the TST following chronic isolation stress in adolescent *mice*, although it did reduce levels of nitrites in their hippocampus [27]. The reduction in the time spent immobile in the TST by *mice* exposed to ISD can be interpreted as an enhanced reactivity of defeated *mice* to the situation of moderate inescapable stress that the TST represents, rather than a reduction of depressive-like behaviour (see a more detailed discussion in [5]). In support of this idea, we have observed in the present study that the time spent immobile correlated positively with the ISI, which was clearly decreased in defeated *mice*. Our results show that 7-NI did not modify the enhanced reactivity of defeated *mice* to the stressful situation of TST, though this compound did reverse the increase in the latency to become immobile induced by ISD.

In the present study we aimed to evaluate the effects of ISD on the cognitive performance of *mice* with the ORT and to explore the potential role of 7-NI in such effects, as previous studies have reported controversial results concerning the consequences of defeat on recognition memory. CSDS was found to reduce the discrimination index in the ORT in some studies [28,29], but not in others [30]. In a previous study, we also failed to observe effects of four episodes of social defeat (on alternate days) in the ORT [31]. In accordance with this, ISD, alone or with 7-NI, did not induce changes in recognition memory in the present study. An important factor in the effects of social defeat in the ORT may be the time elapsed between defeat exposure and testing, as CSDS has been shown to reduce the discrimination index at 7 days (but not at 1 or 21 days) after the last episode of defeat [32]. Future studies should take into account this variable to evaluate the potential role of nNOS in memory recognition, particularly in light of a recent report that 7-NI (80 mg/kg, 4–5 days before ORT) reversed the cognitive deficits observed in a *mouse* model of autism [26].

The most relevant result of the present paper is that blockade of nNOS with 7-NI during episodes of social defeat in late adolescence reversed the effects of ISD on the sensitivity of adult *mice* to cocaine reward. In agreement with previous studies of our laboratory the dose of 1 mg/kg of cocaine is ineffective to induce CPP in non-stressed *mice* [4,5,6,7]. Conversely, *mice* treated with vehicle before each episode of ISD acquired cocaine CPP after conditioning with this subthreshold dose (as reflected by the significant increase in the time spent in cocaine-paired compartment in post-C with respect to pre-C, Figure 4a). The fact that *mice* treated with 7-NI before defeat episodes did not acquire CPP (the same observed in control *mice* without stress exposure) indicates that both doses of this compound prevented the effects of ISD, although it is important to note that there are not significant differences between the CPP score of *mice* exposed to ISD treated with vehicle or 7-NI (Figure 4b). Thus, it seems that inhibition of nNOS with 7-NI reduces, but does not completely block, the effects of ISD on cocaine reward. There are no previous studies on the involvement of the NO pathway in the effects of social defeat on the rewarding properties of cocaine; however, in line with the results observed here, we have previously observed that 7-NI reversed the effects of a similar protocol of ISD on MDMA-induced CPP [12]. In addition, blockade of the NMDA receptors, which are closely related with nNOS inhibition, also reversed the effects of ISD on cocaine reward [11]. nNOS is a downstream signal molecule of NMDA receptor activation. Stimulation of NMDA receptors induces a calcium influx and, in the presence of calmodulin, activates nNOS and the production of NO. In addition, post-synaptically produced NO acts as a retrograde neurotransmitter on pre-synaptic neurons and causes glutamate release, which in turn activates post-synaptic NMDA receptors to increase calcium and further activate nNOS and NO production. This close relationship between NMDA receptors and nNOS can explain why memantine (that blocks NMDA receptors) acts in the same way as 7-NI (which inhibits nNOS), in this case, preventing the effects of social defeat on cocaine-induced CPP. Irrespective of stress exposure, there is experimental evidence that supports a role for nNOS in the addiction-like effects of cocaine in *rats*. Abstinence from cocaine self-administration increases *nNOS* gene and protein expression in the NAcc, but not in the PFC [33], while re-exposure to cocaine-conditioned stimuli following self-administration and extinction increases extracellular glutamate, leading to release of NO in the NAcc core [34]. Moreover, elimination of nNOS-expressing interneurons in the NAcc has been shown to reduce cue-induced cocaine seeking, while stimulation of these interneurons was reported to provoke drug-seeking in the absence of cues [35]. Administration of 7-NI attenuates the symptoms of abstinence induced by cocaine withdrawal and protects against cocaine-induced oxidative stress [36], attenuates CPP and the increase of opioid mu receptors in the NAcc induced by cocaine [37], and reduces the expression of behavioural sensitization and hippocampal synaptic plasticity induced by cocaine [38]. Similarly, the selective nNOS inhibitor L-NPA prevents cocaine CPP [39], undermines the reinstatement of cocaine self-administration induced by cocaine priming or drug-associated cues [33] and, when administered intra-PFC, prevents cocaine-induced presynaptic plasticity in cholinergic neurons of the laterodorsal tegmentum [39]. As a whole, these studies support a role for nNOS in the effects of cocaine in basal conditions and after stress exposure, mainly in the hippocampus and NAcc. Regarding the possible molecular mechanism of action of 7-NI underlying its ability to prevent the long-term effects of stress on cocaine reward, the most probable mechanism is the neuroprotective effect due to the inhibition of nNOS which reduced the neurotoxicity induced by overstimulation of NMDA receptors and nNOS-mediated production of NO (which in turn produces the free radicals responsible for oxidative injury) [36]. In addition, nNOS activation in the hippocampus altered the function of glucocorticoid receptors, an effect that can be reversed by 7-NI [40]. The increase in corticosterone levels induced by stress can activate nNOS through the mineral–corticoid receptors, resulting in the downregulation of hippocampal glucocorticoid receptors and the increase in hypothalamic corticotropin-releasing hormone [41]. On the other hand, 7-NI has antioxidant properties that are independent of nNOS [42].

In the present study, the potent effect of 7-NI in reversing the long-term potentiation of cocaine reward induced by ISD contrasts with the null influence of this compound on the behaviours of defeated *mice* in the short term after the last episode of ISD (anxiety-like symptoms in the EPM, social avoidance in the SIT and stress hyperreactivity in the TST). As mentioned above, some studies have suggested that the increase in anxiety induced by different protocols of stress is related with the inhibition of iNOS rather than the inhibition of nNOS [17,18,19,20,21,27]. Another possible explanation is that the anxiety- and depression-like behaviours induced by ISD were evaluated 24–48 h after the last episode of defeat and thus at the same time point after 7-NI administration. It is relevant to note that the effects of nNOS inhibitors on depression-like behaviour induced by stress depends on several factors, including the type of stressor, the protocol of drug administration and the moment at which the behaviour is evaluated. For example, treatment for 7 days with a daily injection of 7-NI (30 mg/kg) reduced the time spent immobile in the TST and FST of *mice* exposed to CMS 2 and 4 weeks (but not 24 h) after drug administration [23]. Future studies with longer intervals between 7-NI administration/ISD and behavioural evaluation should be performed to determine if anxiety-like symptoms, social avoidance and stress hyperreactivity induced by ISD depend on the integrity of nNOS. It would also be of interest to evaluate the role played by a selective iNOS inhibitor in the short- and long-term effects of ISD.

In conclusion, our results support a specific role for nNOS signalling in the effects of ISD stress on cocaine reward and point to the inhibition of NOS as a therapeutic option to enhance resilience to the effects of social stress on vulnerability to develop cocaine-use disorders.

## 4. Materials and Methods

### 4.1. Subjects

An amount of 44 C57BL/6 male *mice* and 17 OF1 male *mice* (Charles River, France) arrived in the laboratory at 21 days and 42 days of age, respectively. Experimental *mice* (C57BL/6) were grouped (n = 4–5) in plastic cages (25 × 25 × 14.5 cm) while aggressive opponents (OF1) were housed individually in plastic cages (23 × 32 × 20 cm) to induce heightened aggression [43]. *Mice* were maintained under standard laboratory conditions with food and water ad libitum and a reversed light schedule (lights on 19:30–07:30). All experimental protocols were initiated 26 days after the arrival of the *mice* at the laboratory. Procedures involving *mice* and their care were approved by the Ethics Committee of Experimental Research of the University of Valencia (2023-VSC-PEA-0288) and were performed according to Directive 2010/63/EU.

### 4.2. Drugs

Ninety minutes before each episode of defeat, experimental *mice* were injected with 7.25 or 12.5 mg/kg of 7-NI (Merck, Darmstadt, Germany) dissolved in physiological saline (NaCI 0.9%) with DMSO (dimethyl sulfoxide, 2/3 and 1/3 of the total volume, respectively). Control groups non exposed to stress (control) and exposed to stress (vehicle + ISD) were treated with the vehicle used to dissolve 7-NI (2/3 of physiological saline and 1/3 of DMSO). To induce CPP, experimental *mice* were injected with 1 mg/kg of cocaine (Alcaliber Laboratory, Madrid, Spain) dissolved in physiological saline. All compounds were injected intraperitoneally in a volume of 0.01 mL/g of weight. The doses of cocaine and 7-NI was selected on the basis of previous studies carried out in our laboratory ([5,12], respectively).

### 4.3. Experimental Protocols

#### 4.3.1. Intermittent Social Defeat (ISD)

Each experimental *mouse* underwent an agonistic encounter of 25 min (on PND 47, 50, 53 and 56) with an OF1 isolated *mouse*, which defeated the experimental animal. Each encounter consisted of three phases. In the first phase (10 min) an experimental *mouse* (intruder) was introduced into the home cage of an aggressive opponent (resident); the two animals were separated by a wire mesh wall, which protected the intruder from attack but allowed social interaction and threats from the resident. In the second phase (5 min) the wire mesh was removed allowing confrontation between the two *mice*. In the third phase (10 min), the wire mesh was again introduced to separate the two animals but allow for social threats by the resident. All experimental *mice* displayed defeat (a specific posture characterized by an upright submissive position, limp forepaws, upwardly angled head, and retracted ears [44,45]), given that they all faced resident *mice* with high levels of aggression. Intruder *mice* were exposed to a different aggressor *mouse* during each episode of social defeat. The first and fourth episodes were videotaped and later an observer blind to the treatment [46] recorded, with a computerized system (Raton Time 1.0 software; Fixma SL, Valencia, Spain), the time spent in and the latencies of different behavioural categories in the experimental *mice* (avoidance/flee and defence/submission) and in the resident *mice* (threat and attack). The control (non-stressed) group underwent the same protocol in an empty cage (without the presence of a resident *mouse*).

#### 4.3.2. Elevated Plus Maze (EPM)

Experimental *mice* underwent the EPM on PND 57 in order to evaluate the anxiogenic effects of ISD. The apparatus, made of plexiglass, consisted of two open arms and two enclosed arms (30 × 5 cm each one) with a central platform (5 × 5 cm) and was elevated 45 cm above floor level. *Mice* were placed on the central platform facing an open arm and were allowed to explore the maze for 5 min. The behaviour of the *mice* was video recorded and later analysed by an observer blind to the treatment, using a computerized method (Raton Time 1.0 software; Fixma SL, Valencia, Spain). The EPM test is based on the natural aversion of *mice* to open areas and the spontaneous exploratory behaviour they exhibit in novel environments. Anxiety levels are considered to be lower when the measurements in the open arms (time spent in, number of entries and percentages of time and entries) are higher than in the closed arms [47]. Besides these measurements, latency to enter in each arm, the time spent in and number of entries in the central platform and the total number of entries into the arms (a locomotor activity score [48]) were recorded. An arm was considered to have been visited when the animal placed all four paws on it. Measures of open arms (latency (LOA), time spent in (TOA), number of entries (EOA), percentage of time [(open/open + closed) × 100] spent in (%TOA), percentage of entries (%EOA), and total entries (TotalE) into the arms) were taken into account for the statistical analyses.

#### 4.3.3. Social Interaction Test

The effects of ISD on the social behaviour of *mice* was evaluated on PND 57 in the social interaction test. The apparatus used was an open field (37 × 37 × 30 cm) which contained a perforated plexiglass cage (10 × 6.5 × 30 cm) placed in the middle of one wall of the open field. The test has two phases in which the *mouse* is placed in the centre of the open field and allowed to explore it twice for 10 min, under two different experimental conditions: with the perforated plexiglass cage empty (object phase) or containing an unfamiliar OF1 *mouse* (social phase). The perforated cage safeguards the experimental *mouse* from attack in both phases, the time spent in the 8 cm area surrounding the perforated cage—the interaction zone—was registered and automatically sent to a computer using the Ethovision 2.0 software package (Noldus, Wageningen, The Netherlands). Between both phases, the experimental *mouse* was returned to its home cage for 2 min. An index of social interaction (ISI) was obtained [time spent in the interaction zone during the social phase/(time spent in the interaction zone during the social phase + time spent in the interaction zone during the object phase)] [49]. The ISI is commonly used as an index of social preference or avoidance [50].

#### 4.3.4. Object Recognition Test (ORT)

To evaluate the effects of ISD on relational memory we used the ORT [48,51]. This test consists of three phases. In the first phase (habituation), *mice* are placed in the centre of a box and allowed to explore it for two minutes (this phase was performed on PND 57). Twenty-four hours later, in the second phase (training session, T1) *mice* are once again placed (for five minutes) in the box, with two stones placed in opposite corners. Afterwards, *mice* are returned to their home cage for one minute (memory retention interval). In the last phase (test session, T2) one of the stones is replaced with a small non-toxic plastic toy. Subsequently, the *mice* are placed once again in the box for another five minutes. The following measures are recorded using a computerized system (Raton Time 1.0 software; Fixma SL, Valencia, Spain): total time spent exploring the two stones in T1; and time spent exploring the stone and the toy in T2. These measures are used to calculate the discrimination index [DI = (Tnovel − Tfamiliar)/(Tnovel + Tfamiliar) × 100%], as previously described [31].

#### 4.3.5. Tail Suspension Test (TST)

On PND 58, we investigated whether ISD modified the behavioural variable of immobility in the TST, which is considered to represent despair [52]. This test is based on the observation that when *mice* are placed in an inescapable and uncontrollable stressful situation, such as being hung by their tail, after initial escape attemps, they develop an immobile posture [53]. The TST has been used as a measure of behavioural depression because animals treated with antidepressant drugs engage in escape-directed behaviours for more time [52]. *Mice* were suspended by the tail (using adhesive tape) from a hook for 6 min [54] and their behaviour was video recorded and later analyzed by an observer blind to the treatment, using a computerized method (Raton Time 1.0 software; Fixma SL, Valencia, Spain) to measure the total time spent immobile (Immobility) and the latency to become immobile (LImmobility).

#### 4.3.6. Conditioned Place Preference (CPP)

The long-term effects of ISD on cocaine reward were evaluated three weeks after the last episode of social defeat (on PND 77) using the CPP procedure. Eight identical plexiglass boxes with two equal-sized compartments (30.7 cm long × 31.5 cm wide × 34.5 cm high) with different coloured walls (black vs. white) and distinct floor textures (fine vs. wide grid), separated by a gray central area (13.8 cm long × 31.5 cm wide × 34.5 cm high) were used. The boxes, equipped with infrared light beams to record the position of the animal, were controlled by three IBM PC computers using MONPRE 2Z 1.0 software (Cibertec SA, Madrid, Spain). *Mice* underwent an unbiased CPP procedure with three phases (see details in [55]). In the first phase (pre-conditioning, pre-C), the time spent by the animal in each compartment was recorded for 15 min over 3 consecutive days. Animals showing a strong unconditioned aversion or a preference for a given compartment (less than 33% or more than 66% of the total session time, respectively) were excluded from the study (n = 3). In the second phase (conditioning, 4 days) *mice* received saline and were confined to the vehicle-paired compartment for 30 min, and, after an interval of 4 h, were injected with 1 mg/kg of cocaine and were immediately confined to the drug-paired compartment for 30 min. During the third phase (post-conditioning, post-C), the time spent by the untreated *mice* in each compartment during a 15 min period was again recorded.

### 4.4. Experimental Design

**Figure 5:**
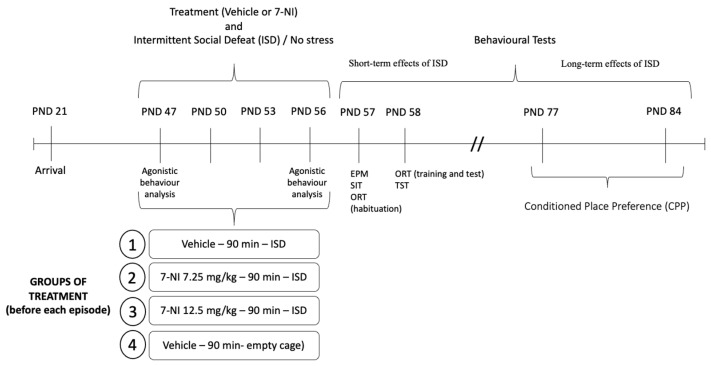
Timetable and experimental design. PND: post-natal day; 7-NI: 7-nitroindazole; EPM: elevated plus maze; SIT: social interaction test; ORT: object recognition test; TST: tail suspension test.

According to the treatment received, four groups of *mice* were used (Figure 5): a control group (vehicle + no stress), a stressed group (vehicle + SD), and two stressed groups treated with 7-NI (7.25 or 12.5 mg/kg) before each episode of SD (7-NI7 + SD and 7-NI12 + SD). Shortly after the last episode of SD (24/48 h) the *mice* were tested in the EPM, SIT, ORT and TST. Behavioural tests took place in a dimly illuminated experimental room during the dark period (08.30–16.30) and were transported to this room 1 h prior to testing in order to facilitate adaptation. The order of the behavioural tests was based on a previous study, according to the degree of stress that the same tests had induced in the *mice*; in this way, we aimed to prevent previous experience in a test from affecting the *mouse*’s performance in subsequent tests [5]. As the open arms measurements are very sensitive to environmental conditions and prior manipulation of the animal, we decided to perform the EPM first. Following the same logic, the TST was performed last because it is the most stressful test. Subsequently, after an interval of 3 weeks, all *mice* were conditioned in the CPP paradigm with a subthreshold dose of cocaine (1 mg/kg).

### 4.5. Statistical Analyses

Sample distributions were assessed for normality (Kolmogorov–Smirnov test) and homogeneity (Levene’s test). In the case of unequal variance, we used the Welch ANOVA as alternative to the classic ANOVA. Post-hoc comparisons after ANOVA were performed with Bonferroni.

Regarding behaviour during the episodes of defeat, differences between groups and between the first and fourth episodes were evaluated with mixed ANOVAs, using the treatment (with three levels, vehicle + SD, 7-NI7 + SD and 7-NI12 + SD) and days variables (first and fourth episode of defeat).

The data obtained in the tests performed shortly after ISD and the CPP scores (time spent in post-C minus time spent in pre-C) were analysed by one-way ANOVA with a between variable—treatment—with four levels (vehicle + no stress, vehicle + SD, 7-NI7 + SD and 7-NI12 + SD). The following measurements were included in the statistical analyses: TOA, EOA, LOA, %TOA, %EOA and TotalE in the EPM, ISI, Immobility and LImmobility in TST, recognition Index in the ORT and CPP score. In addition, the data regarding the time spent in the drug-paired compartment were analysed with a two-way ANOVA with a between variable—treatment—with four levels (vehicle + no stress, vehicle + SD, 7-NI7 + SD and 7-NI12 + SD) and a within variable—days—with two levels (pre-C and post-C).

Pearson correlation tests were used to determine relationships among the different behaviours during the defeat episodes and the performance of stressed *mice* in each behavioural test. In the case of significant correlations, we also performed lineal regression analysis. All statistical analyses were performed with the SPSS program, version 29.0.2.0.

## Figures and Tables

**Table 1:** Behavioural categories evaluated during episodes of social defeat.

**Experimental** **Animals**	**Avoidance/** **Flee**		**Latency Avoid/Flee**		**Defence/** **Submission**		**Latency** **Defence/Subm.**	
**Encounter**	**1**	**4**	**1**	**4**	**1**	**4**	**1**	**4**
Defeated *mice* treated with VEH	117.39	168.72	[26.52	5.15]	15.64	20.86	40.71	73.2
(n = 10)	±31.24	±22.61	±13.71	±1.53	±3.23	±5.38	±17.17	±34.82
Defeated *mice* treated with 7-NI7	142.87	155.49	4.64	4.32	9.5	15.39	74.67	37.86
(n = 12)	±20.28	±21.24	±2.07	±1.31	±2.43	±3.89	±27.12	±12.81
Defeated *mice* treated with 7-NI12	96.5	125.21	[2.69	2.67] *	14.27	17.54	65.84	31.75
(n = 12)	±17.73	±24.63	±0.55	±0.91	±4.32	±3.38	±26.53	±12.03
**Aggressive** **Opponents**	**Threat**		**Latency** **Threat**		**Attack**		**Latency** **Attack**	
**Encounter**	**1**	**4 #**	**1**	**4**	**1**	**4**	**1**	**4**
Confronted with VEH-treated *mice*	[15.89	10.32]	23.48	43.56	40.96	36.47	22.28	6.98
(n = 10)	±2.77	±2.17	±13.26	±19.25	±5.18	±7.7	±12.79	±2.06
Confronted with 7-NI7 treated *mice*	14.01	8.32	15.26	25.39	35.1	29.09	3.78	3.73
(n = 12)	±3.33	±1.84	±7.1	±9.93	±3.69	±2.9	±0.98	±1.12
Confronted with 7-NI12 treated *mice*	[6.84	6.4] *	22.46	41.32	31.18	26.04	2.67	4.6
(n = 12)	±1.53	±1.59	±8.35	±15.65	±4.35	±5.22	±0.55	±2.27

Mean time (±SEM) spent in different behavioural categories by experimental animals (avoidance/flee, defence/submission and latency to initiate these behaviours) treated with vehicle (VEH) or 7-nitroindazole at the doses of 7.5 (7-NI7) or 12.5 (7-NI12) mg/kg and by aggressive opponents confronted by them (threat, attack and latency to initiate these behaviours) in the first (1) and fourth (4) agonistic encounter. As interaction days X treatment was not significant, squared brackets [] indicate the treatments that differ significantly considering the data from the first and fourth episodes together. * *p* < 0.05, significant difference with respect to *mice* treated with vehicle (7-NT12 + SD vs. vehicle + SD group); # *p* < 0.05, significant difference with respect to the first encounter.

**Table 2:** Pearson correlations between behavioural categories shown by experimental and opponent *mice* during agonistic encounters.

	Threat 1	Threat 4	LThreat 1	LThreat 4	LAttack 1	Av/Flee 1	Av/Flee 4	LAv/Flee 1	LAv/Flee 4	Def/S 1	Def/S 4	LDef/S 1	LDef/S 4
**Threat 1**		ns	ns	ns	ns	*r* = −0.357; R^2^ = 0.127	ns	ns	ns	ns	ns	ns	ns
**Threat 4**	ns		ns	ns	ns	ns	*r* = −0.444; R^2^ = 0.197	ns	ns	ns	*r* = 0.613; R^2^ = 0.375	ns	ns
**LThreat 1**	ns	ns		ns	*r* = 0.638; R^2^ = 0.407	ns	ns	*r* = 0.58; R^2^ = 0.336	ns	ns	ns	*r* = 0.461; R^2^ = 0.213	ns
**LThreat 4**	ns	ns	ns		ns	ns	ns	ns	ns	ns	*r* = −0.357; R^2^ = 0.128	ns	*r* = 0.516; R^2^ = 0.266
**LAttack 1**	ns	ns	0.001	ns		ns	ns	*r* = 0.937; R^2^ = 0.879	ns	ns	ns	ns	ns
**Av/Flee 1**	0.05	ns	ns	ns	ns		ns	*r* = −0.36; R^2^ = 0.129	ns	*r* = −0.458; R^2^ = 0.210	ns	ns	ns
**Av/Flee 4**	ns	0.01	ns	ns	ns	ns		ns	ns	ns	*r* = −0.731; R^2^ = 0.535	ns	*r* = 0.423; R^2^ = 0.179
**LAv/Flee 1**	ns	ns	0.001	ns	0.01	0.05	ns		ns	ns	ns	ns	ns
**LAv/Flee 4**	ns	ns	ns	ns	ns	ns	ns	ns		ns	ns	ns	ns
**Def/S 1**	ns	ns	ns	ns	ns	0.01	ns	ns	ns		ns	*r* = −0.498; R^2^ = 0.248	ns
**Def/S 4**	ns	0.001	ns	0.05	ns	ns	0.01	ns	ns	ns		ns	*r* = −0.485; R^2^ = 0.235
**LDef/S 1**	ns	ns	0.01	ns	ns	ns	ns	ns	ns	0.01	ns		ns
**LDef/S 4**	ns	ns	ns	0.01	ns	ns	0.05	ns	ns	ns	0.01	ns	

Behavioural measurements: Threat, latency of threat, latency of attack, avoidance/flee, latency of avoidance/flee, defence/submission, latency of defence/submission. 1: first episode of defeat; 4, fourth episode of defeat. Upper part: *r* and R^2^ values for significant correlations, ns: no significant correlation. Lower part: level of significance. Only measurements with significant correlations were shown.

**Table 3:** Pearson correlations between behaviours in episodes of defeat and behavioural tests.

	Threat 1	Attack 1	Attack 4	LAttack 1	Av/Flee 4	LAv/Flee 1	Def/S 4	Immob	LImmob	TOA	%TOA	EOA	%EOA	ISI	CPP
**Threat 1**		ns	ns	ns	ns	ns	ns	ns	ns	ns	ns	ns	ns	*r* = −0.356; R^2^ = 0.126	ns
**Attack 1**	ns		ns	ns	ns	ns	ns	ns	ns	*r* = 0.361; R^2^ = 0.130	*r* = 0.371; R^2^ = 0.137	*r* = 0.361; R^2^ = 0.130	ns	ns	ns
**Attack 4**	ns	ns		ns	ns	ns	ns	ns	ns	*r* = 0.401; R^2^ = 0.161	*r* = 0.363; R^2^ = 0.131	ns	*r* = 0.342; R^2^ = 0.117	*r* = 0.369; R^2^ = 0.136	ns
**LAttack 1**	ns	ns	ns		ns	*r* = 0.937; R^2^ = 0.879	ns	ns	*r* = 0.507; R^2^ = 0.257	ns	ns	ns	ns	ns	ns
**Av/Flee 4**	ns	ns	ns	ns		ns	*r* = −0.731; R^2^ = 0.535	ns	ns		*r* = 0.366; R^2^ = 0.134	ns	ns	ns	ns
**LAv/Flee 1**	ns	ns	ns	0.001	ns		ns	ns	*r* = 0.475; R^2^ = 0.225	ns	ns	ns	ns	ns	ns
**Def/S 4**	ns	ns	ns	ns	0.001	ns		*r* = 0.345; R^2^ = 0.119	ns	*r* = −0.496; R^2^ = 0.246	*r* = −0.431; R^2^ = 0.186	*r* = −0.424; R^2^ = 0.179	ns	ns	*r* = 0.371; R^2^ = 0.138
**Immob**	ns	ns	ns	ns	ns	ns	0.05		ns	ns	ns	ns	ns	*r* = 0.443; R^2^ = 0.196	ns
**L Immob**	ns	ns	ns	0.01	ns	0.01	ns	ns		ns	ns	ns	ns	ns	ns
**TOA**	ns	0.05	0.05	ns	0.05	ns	0.01	ns	ns		*r* = 0.96; R^2^ = 0.922	*r* = 0.878; R^2^ = 0.771	*r* = 0.821; R^2^ = 0.674	ns	ns
**%TOA**	ns	0.05	0.05	ns	0.05	ns	0.05	ns	ns	0.001		*r* = 0.852; R^2^ = 0.725	*r* = 0.888; R^2^ = 0.788	ns	ns
**EOA**	ns	0.05	ns	ns	ns	ns	0.05	ns	ns	0.001	0.001		*r* = 0.825; R^2^ = 0.681	*r* = −0.381; R^2^ = 0.145	ns
**%EOA**	ns	ns	0.05	ns	ns	ns	ns	ns	ns	0.001	0.001	0.001		ns	ns
**ISI**	0.05	ns	0.05	ns	ns	ns	ns	0.01	ns	ns	ns	0.05	ns		ns
**CPP**	ns	ns	ns	ns	ns	ns	0.05	ns	ns	ns	ns	ns	ns	ns	

Behavioural measurements: Threat, attack, latency of attack, avoidance/flee, latency of avoidance/flee, defence/submission, time of immobility in the TST, latency of immobility in the TST, measurements of the open arms (OA) in the EPM (time, percentage of time, entries and percentage of entries), index of social interaction (ISI), CPP score. 1: first episode of defeat; 4, fourth episode of defeat. Upper part: *r* and R^2^ values for significant correlations, ns: no significant correlation. Lower part: level of significance. Only measurements with significant correlations were shown.

## Data Availability

The raw data supporting the conclusions of this article will be made available by the authors on request.

## Supplementary Materials

The following supporting information can be downloaded at https://www.mdpi.com/article/10.3390/ph17091203/s1, Figure S1: Role of 7-nitroindazole in the effects of intermittent social defeat (SD) on the behaviour of *mice* in the object recognition test.
