# Supplementary figures and images for: Inhibition of Nitric Oxide Synthesis Prevents the Effects of Intermittent Social Defeat on Cocaine-Induced Conditioned Place Preference in Male Mice
Source: Pharmaceuticals (Basel). 2024 Sep 12;17(9):1203. doi: 10.3390/ph17091203 (PMC11435249; doi:10.3390/ph17091203)

# Object Recogn.

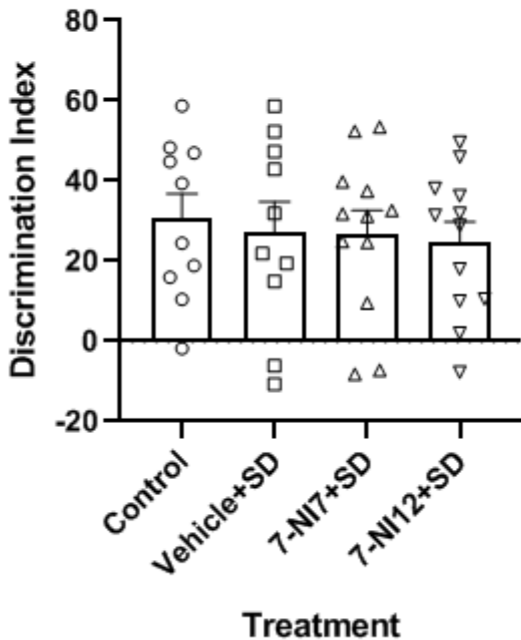

Supplement: Supplementary file 1 [file pharmaceuticals-17-01203-s001.zip › pharmaceuticals-3173923-supplementary.pdf]
